# Simulated Solute Tempering 2: An Efficient and Practical Approach to Protein Conformational Sampling and Binding Events

**DOI:** 10.1021/acs.jctc.5c00950

**Published:** 2025-10-27

**Authors:** Dirk Stratmann, Gautier Moroy, Pierre Tuffery, Samuel Murail

**Affiliations:** † 555089 Université Paris Cité, CNRS UMR 8251INSERM ERL U1133, Unité de Biologie Fonctionnelle et Adaptative, BFA, F-75013 Paris, France; ‡ Sorbonne Université, Faculté des Sciences et Ingénierie, UFR 925, F-75252 Paris, France

## Abstract

Molecular dynamics (MD) simulations are powerful tools for studying the movement and interactions of molecules, but they can be computationally expensive, especially for large biomolecules like proteins. This is problematic because accurately simulating the motions of these molecules is key to understanding their functions. Enhanced sampling methods, such as Simulated Tempering (ST), temperature Replica Exchange Molecular Dynamics (REMD), and Replica Exchange with Solute Tempering (REST and REST2), have been developed to overcome this challenge by improving the efficiency of MD simulations. This work presents a new enhanced sampling method called Simulated Solute Tempering 2 (SST2) that builds upon the strengths of ST and REST2. SST2 selectively scales the interactions inside a biomolecule and with its surrounding environment, effectively accelerating the exploration of its different structural states and their stabilities at various temperatures. SST2 is tested on three different systems (chignolin CLN025, Trp-Cage, and a protein–peptide complex, p97/PNGase) and is found to achieve comparable or superior sampling efficiency to ST, SST1, and REST2 while requiring fewer temperature rungs. Notably, SST2 is particularly well-suited for investigating large biomolecular systems, making it a valuable tool for studying a wide range of biomolecular processes, from protein folding to ligand binding.

## Graphical abstract

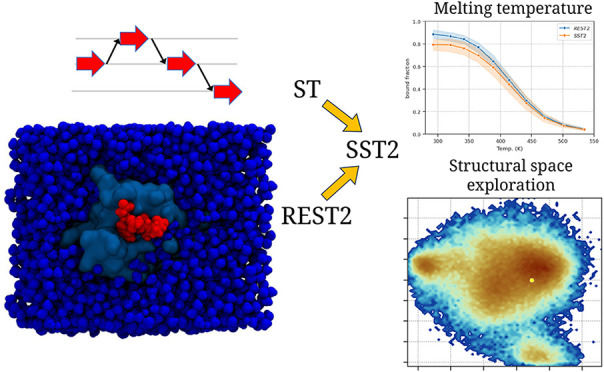

## Introduction

Recent breakthroughs in deep learning, exemplified by AlphaFold 2,^1^ have led to unprecedented success in protein structure prediction. This progress represents a crucial step toward a deeper understanding of protein functions. Derivatives of AlphaFold 2, such as Colabfold^2^ and AlphaFold-Multimer,^3^ have further extended the scope of protein structure prediction by allowing the modeling of protein complexes and multimers. In the context of protein complexes, the specific case of protein–peptide complexes has demonstrated that these methods can accurately predict the structure of protein–peptide complexes for 60 to up to 90% of complexes.^4,5^

However, predicting a static protein structure is only one part of the story, and a nuanced understanding of protein function requires a thorough exploration of protein dynamics and the multiple conformations that proteins can adopt. Molecular dynamics (MD) simulations, based on Newton’s equations of motion, provide a means of predicting the temporal evolution of biomolecular systems.^6–8^ Despite their potential, MD simulations are often limited by their computational cost when capturing the time scales of critical conformational changes, particularly at the microsecond scale and beyond.

During MD simulations, proteins frequently become trapped in the local minima of the free energy landscape, which hinders the exploration of conformational diversity. To overcome these limitations, several enhanced sampling techniques have been developed, such as temperature Replica Exchange Molecular Dynamics (for simplicity, REMD will be used as temperature REMD)^9^ and Simulated Tempering (ST).^10,11^ REMD runs parallel simulations at different temperatures, allowing periodic exchanges between replicas based on a Metropolis criterion. In contrast, ST involves a single simulation with periodic updates of the temperature, guided by a Metropolis criterion that takes into account the potential energy and weights.

Although REMD and ST are effective in solving local minima problems, their efficiency depends on the size of the simulated system, as the optimal number of replicas or temperature ladders increases greatly with the degrees of freedom of the simulated system. For large systems, the number of replicas required to achieve efficient temperature sampling is too high to be practical.

In order to address these challenges, Liu et al. introduced Replica Exchange with Solute Tempering (REST).^12^ The REST method separates the system into solute and solvent, allowing the solute to ascend the temperature ladder while *maintaining* the solvent at ambient temperature (this is a physical approximation; the system is in fact warmed up; however, in combination with the modified potential energy function, the solvent is considered to be maintained at ambient temperature). However, the REST method has been demonstrated to be less efficient than REMD for larger systems, such as Trp-Cage and β hairpin.^13^ The subsequent REST2 method^14^ is a pure Hamiltonian REMD. This ensures that all replicas run at the same temperature, but evolve with different Hamiltonians. Only the solute–solute and solute–solvent interactions are modified to mimic the effect of elevated temperature. The main difference between REST and REST2 is the scaling of the solute–solvent term. REST2 employs a weaker solute–solvent interaction at *“high temperature”*, which favors the sampling of folded conformations under these conditions. Since then, REST2 has become a benchmark for conformational sampling of small proteins in different contexts.^15–17^ It has been implemented in popular MD simulation software like Gromacs and NAMD.^18–20^

In addition to its efficiency, REST2 has the advantage of simulating solvents at ambient temperature, mitigating the challenges associated with the limited parameterization of water molecules at elevated temperatures. This feature, demonstrated by Stirnemann and Sterpone, allows REST2 to more accurately reconstruct the thermal stability of proteins compared to REMD.^18^

It is worth noting that replica exchange, which depends on the potential energy of two simulations, has a lower probability of exchange compared to ST, resulting in a lower rate of traversal of energy space.^21^ In addition, Rosta and Hummer^22^ have shown that running multiple ST simulations in parallel without communication is as efficient as running REMD simulations with communication for replica exchange. The need for communication between replicas implies the utilization of distributed computations on CPU or GPU nodes that exhibit identical computational performances. As a result, both REMD and REST2 require specific and significant computing power. This can lead to significant performance limitations, particularly in scenarios where the CPU/GPU node park is heterogeneous. If the computational performance varies between the ladders, a simulation may need to wait for others to complete before the replica exchange is attempted and the calculation continues.

The first version of the Simulated Solute Tempering (SST1)^23^ method was developed as a combination of ST and REST. Like REST, SST1 allows a reduced number of replicas, and compared to ST, SST1 shows the highest round-trip rate and the highest sampling speed on an alanine octapeptide.^23^ However, SST1 suffers from the same weaknesses as the first version of REST, and SST1 may be limited for the folding of larger systems with low exchange between folded and extended forms of proteins involving large conformational changes during folding.

In response to these challenges, we present a novel algorithm, Simulated Solvent Tempering 2 (SST2), designed to overcome the limitations of existing methods. By exploiting the strengths of ST and REST2, SST2 aims to achieve both efficient sampling and a straightforward implementation.

To assess the performance of SST2, extensive MD simulations were performed on three different systems: two *toy systems*, chignolin CLN025 and Trp-Cage, and a protein–peptide complex, PNGase, in complex with the p97 peptide. Chignolin CLN025 and Trp-Cage are commonly used to benchmark protein folding methods.^18,24–28^ CLN025 is a β hairpin, and Trp-Cage is an α helix. For the two *toy systems*, SST2 conformational space sampling capabilities have been systematically compared to ST, SST1, and REST2. The ability of SST2 to reconstruct the thermal stability curve of the simulated peptides has been investigated, and the effect of the reference temperature on the efficiency of SST2 has been studied. We also focused on the effect of *cis-trans* isomerization of proline residues on sampling efficiency and how to improve it.

Finally, to assess the algorithm sampling efficiency in ligand binding events and to study larger systems, whose size and complexity would prevent the use of REMD or ST, we applied SST2 to the peptide–protein complex p97/PNGase and compared its performance with that of REST2.

## Results

### CLN025 and Trp-Cage Simulations

As the first evaluation of the SST2 method, we performed explicit solvent simulations of two protein toy systems, chignolin CLN025 and Trp-Cage, starting from both folded (PDB IDs: 5AWL and 1L2Y) and unfolded conformations. For each system, we performed ST, SST1, REST2, and SST2 simulations (Table 1). ST simulations were performed with 20 temperature rungs between 280 and 500 K, while SST2 simulations used 10 λ rungs corresponding to effective solute–solute temperatures between 280 and 540 K at a reference solvent temperature of 300 K. Each trajectory was propagated for 10 μs (CLN025) or 40 μs (Trp-Cage), with four independent replicas for the folded and unfolded states.

**Table 1:** List of Simulations^a^

name	protein	starting form	length (μs)	friction (ps^–1^)	sampling method	min. temp. (K)	max. temp. (K)	ref. temp. (K)	#rungs	Pro ω scaling	#replicas
*F*_*ST*_	CLN025	5awl	10.0	10.0	ST	280	500		20		4
*U*_*ST*_	CLN025	unfolded	10.0	10.0	ST	280	500		20		4
*F*_*SST*1_	CLN025	5awl	10.0	10.0	SST1	280	500	300	10		4
*U*_*SST*1_	CLN025	unfolded	10.0	10.0	SST1	280	500	300	10		4
*F*_*REST*2_	CLN025	5awl	1.0	1.0	REST2	280	540	300	10	yes	4
*U*_*REST*2_	CLN025	unfolded	1.0	1.0	REST2	280	540	300	10	yes	4
*F*	CLN025	5awl	10.0	1.0	SST2	280	540	300	10	yes	4
*U*	CLN025	unfolded	10.0	1.0	SST2	280	540	300	10	yes	4
*F*_350 *K*_	CLN025	5awl	10.0	1.0	SST2	280	540	350	10	yes	4
*F*_6 *rungs*_	CLN025	5awl	10.0	1.0	SST2	280	540	300	6	yes	4
*F*_*big box*_	CLN025	5awl	10.0	1.0	SST2	280	540	300	10	yes	2
*F*_*ST*_	Trp-Cage	1l2y	40.0	10.0	ST	280	500		20		4
*U*_*ST*_	Trp-Cage	unfolded	40.0	10.0	ST	280	500		20		4
*F*_*SST*1_	Trp-Cage	1l2y	20.0	10.0	SST1	280	500	300	10		4
*U*_*SST*1_	Trp-Cage	unfolded	20.0	10.0	SST1	280	500	300	10		4
*F*_*REST*2_	Trp-Cage	1l2y	2.0	1.0	REST2	280	540	300	10	yes	2
*U*_*REST*2_	Trp-Cage	unfolded	2.0	1.0	REST2	280	540	300	10	yes	2
*F*	Trp-Cage	1l2y	40.0	1.0	SST2	280	540	300	10	yes	2 (1,2)
*F*	Trp-Cage	1l2y	40.0	1.0	SST2	280	540	300	10	no	2 (3,4)
*U*	Trp-Cage	unfolded	40.0	1.0	SST2	280	540	300	10	yes	2 (1,2)
*U*	Trp-Cage	unfolded	40.0	1.0	SST2	280	540	300	10	no	2 (3,4)
*F*_350 *K*_	Trp-Cage	1l2y	40.0	1.0	SST2	280	540	350	10	no	4
*F*_280–700 *K*_	Trp-Cage	1l2y	20.0	1.0	SST2	280	700	300	14	yes	4
*F*_*REST*2_	PNGase-p97	2hpl	1.0	1.0	REST2	280	700	320	10		4
*U*_*REST*2_	PNGase-p97	2hpj + linear peptide	1.0	1.0	REST2	280	700	320	10		4
*F*	PNGase-p97	2hpl	20.0	1.0	SST2	280	700	320	10		4
*U*	PNGase-p97	2hpj + linear peptide	20.0	1.0	SST2	280	700	320	10		4

a PDB reference ID structures for chignolin CLN025, Tryptophan Cage, PNGase-p97, and PNGase in free form are 5awl,^29^ 1l2y,^30^ 2hpl, and 2hpj,^31^ respectively. All simulations were conducted with an exchange time of 2.0 ps.

For SST1, the simulation lengths were 10 μs (CLN025) and 20 μs (Trp-Cage), while REST2 simulations were run for 1 μs × 10 replicas (CLN025) and 2 μs × 10 replicas (Trp-Cage), using the same λ ladder as SST2. All simulations were performed with the Amber14SB force field, TIP3P water, and a 4 fs integration time step with hydrogen mass repartitioning. Exchange attempts were carried out every 2 ps. More details on the simulation setup can be found in the Materials and Methods section.

#### Transition Acceptance Ratio

With 10 λ rungs, SST2 simulations showed a high acceptance ratio energy ( $E_{\mathrm{pp}}+0.5\sqrt{\beta_{\mathrm{ref}}/\beta_{M}}E_{\mathrm{pw}}$ ) overlap for CLN025 (Figure 1B) and Trp-Cage (Figure S1B), resulting in average probabilities of λ transition of about 84% and 66% for CLN025 and Trp-Cage, respectively (Figures 1A and S1A). During the ST simulations of CLN025 and Trp-Cage, we doubled the number of temperature rungs, resulting in lower transition probabilities of about 47 and 31%, respectively (Figures S3A and S4A).

**Figure 1:**
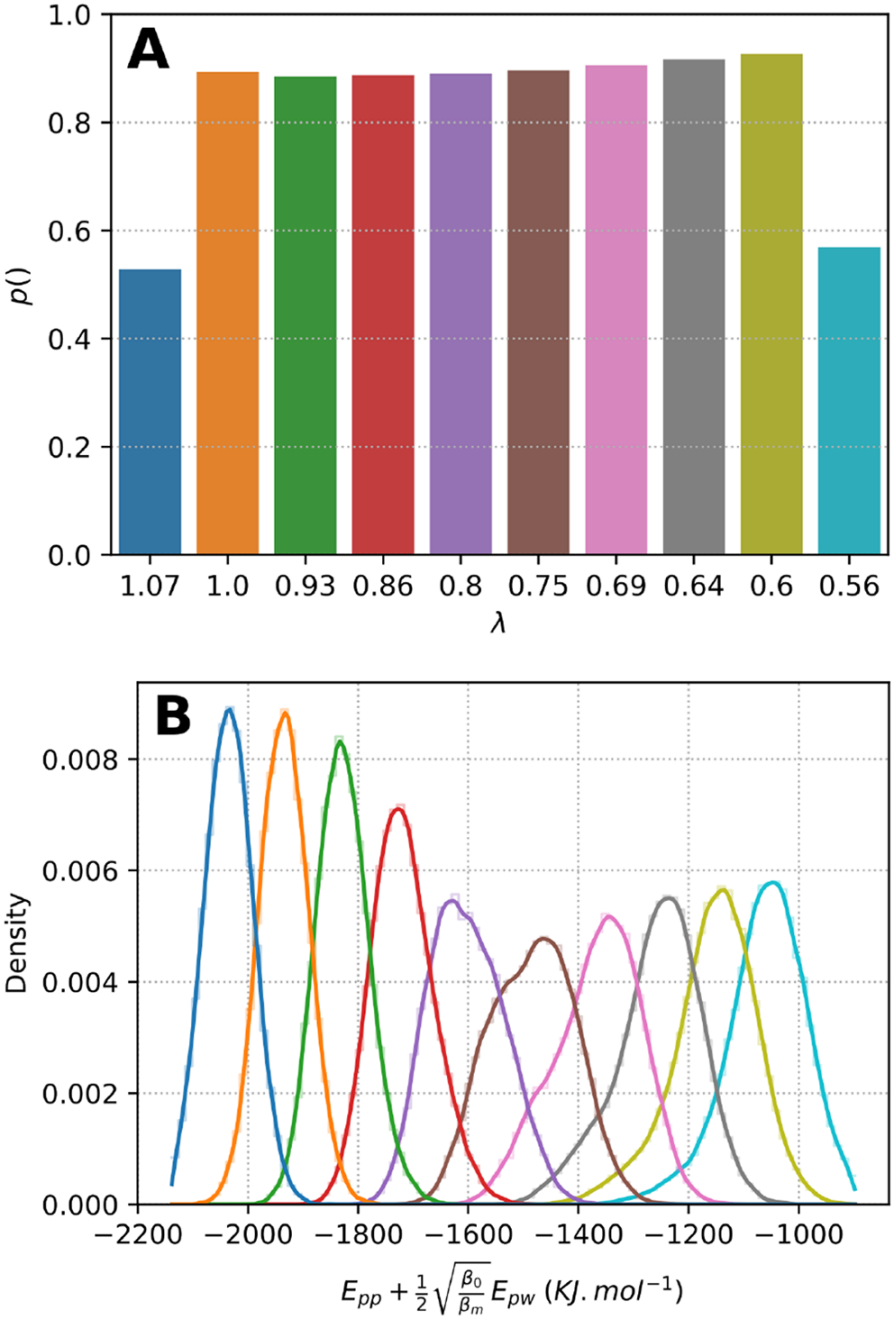
Probability of transition and distribution of the SST2 energy as a function of λ_*i*_ for simulation CLN025 F(1). (A) Probability of transition at each λ rung. (B) Distribution of acceptance ratio energy $E_{\mathrm{pp}}+0.5\sqrt{\beta_{\mathrm{ref}}/\beta_{m}}E_{\mathrm{pw}}$ for each λ; the color code for the λ value is identical to that of panel A. The maximum λ value of 1.07 corresponds to a solute–solute temperature of 280 K, while the minimum λ value of 0.56 corresponds to a temperature of 540 K.

For CLN025, we additionally investigated the effect of the number of rungs on the probability of transition and sampling by performing SST2 simulations of the folded state of CLN025 with only 6 λ rungs (F_6 rungs_). This subset showed a reduced overlap in the acceptance ratio energy (Figure S2B), resulting in a transition probability of about 44% (Figure S2A). Remarkably, for CLN025, the SST2 simulations achieved a similar order of transition probability as the 20 temperature rungs of the ST simulations, despite using only 6 λ rungs.

With 10 λ rungs, the REST2 simulations showed lower acceptance ratios of 32% and 23% for CLN025 and Trp-Cage, respectively. REST2 simulations showed a more than 2-fold lower acceptance ratio, which was expected as two consecutive replicas must be compatible to be swapped. For the SST1 simulations, the acceptance ratios were of the same order of magnitude as those for the SST2 simulations, with 75% and 58% for CLN025 and Trp-Cage, respectively.

#### Dependence of the Folding State on the λ Value

The coevolution of the RMSD and λ values during a subset of CLN025 *F*(1) (the number in parentheses indicates the replica number) simulation is shown in Figure 2. The RMSD of the backbone atoms of CLN025 to the 5awl structure oscillated between the values around 0.1 nm in the folded state and 0.5 nm in the unfolded state (Figure 2A). This RMSD was correlated with the λ value of the SST2 simulation; as the temperature increased or λ decreased, the RMSD of CLN025 increased (Figure 2). The correlation between the CLN025 RMSD and λ values was consistent for the other SST2, SST1, ST (Figures S5 and S6), and REST2 simulations (Figures S7 and S8). The same observation was made for Trp-Cage (Figures S11, S10, S9, and S12). Similar to ST, SST1, and REST2, SST2 simulations showed a correlation between the folding state of the protein and the temperature or λ values of the ladder.

**Figure 2:**
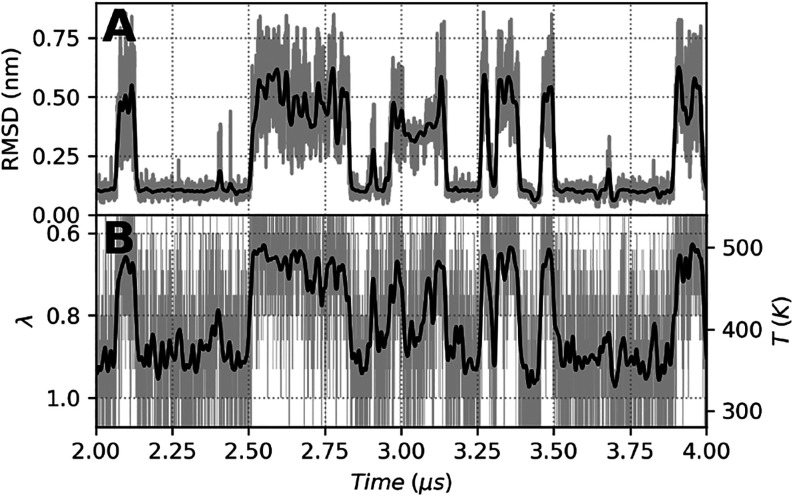
Coevolution of RMSD and λ values during a subset of the simulation CLN025 F(1). (A) RMSD of the backbone atoms of chignolin to the 5awl structure. (B) λ evolution as a function of time; the equivalent solute–solute temperature is indicated on the right *y-*axis. Note that the λ-axis is inverted to highlight the correlation between the RMSD and λ. For clarity, only a 2.0 μs subset of the *F*(1) simulation out of 10.0 μs is shown. For both panels, the raw RMSD and λ values are displayed as gray lines, and a smooth Gaussian filter with σ = 20 applied to the RMSD and λ values is displayed as a black line.

#### Effect of Proline *cis-trans* Transition on Sampling

Proline residues occasionally undergo *cis-trans* transitions of the ω dihedral angle, which can significantly influence protein conformational sampling. In some simulations, such as CLN025 *F*_6;rungs_(1), the transition to the *cis* state trapped the protein in an unfolded conformation for several microseconds (Figure 3). This illustrates how rare events involving proline isomerization can strongly bias the folding dynamics. To avoid such artifacts, we excluded simulation frames containing proline in the *cis* state from our comparative efficiency analyses. We further verified that this issue can be effectively addressed either by excluding the ω dihedral angles from the solute-scaled intramolecular energy term, thereby preventing the transition, or by raising the maximum temperature (e.g., up to 700 K) to accelerate isomerization. Both strategies preserve the reliability of fold–unfold sampling in SST2.

**Figure 3:**
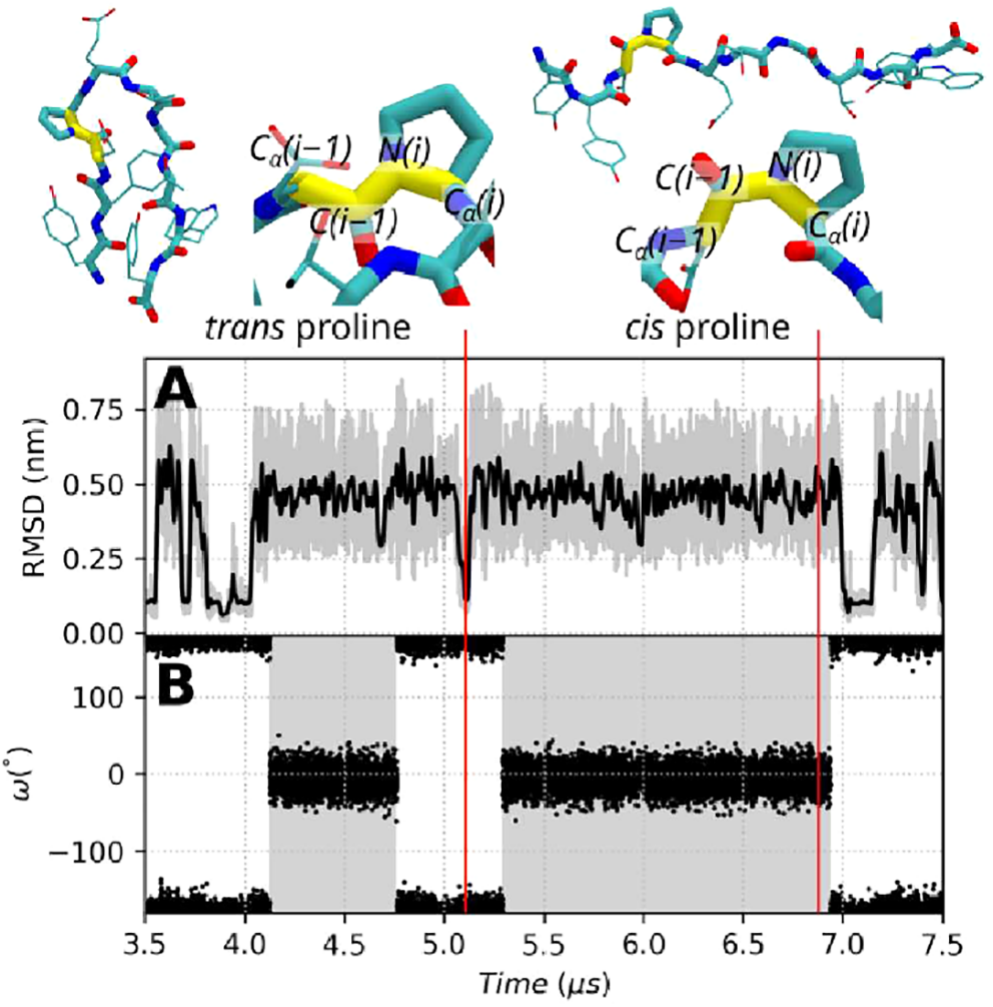
Coevolution of the RMSD and Proline 4 ω dihedral angle during a subset of the simulation CLN025 *F*_6 rungs_(1). (A) RMSD of the backbone atoms of chignolin to the 5awl structure. Raw RMSD values are displayed as gray lines, and a smooth Gaussian filter with σ = 20 applied to the RMSD and λ values is displayed as a black line. (B) Proline 4 ω dihedral angle as a function of time. A gray background highlights when the ω dihedral angle is in the *cis* state. The ω angle is defined as the dihedral angle between the C_α(*i*–1)_–*C*_(*i*–1)_–*N*_(*i*)_–*C*_α(*i*)_ atoms, where *i* is the proline residue index. For clarity, only a 4.0 μs subset of the *F*_6 rungs_(1) simulation out of 10.0 μs is shown.

Further details, including additional Trp-Cage and high-temperature simulations, are provided in the Supporting Information.

#### Fold/Unfold Transitions

The RMSD oscillation of CLN025 and Trp-Cage appears to be on the same order for the ST, SST1, SST2, and REST2 simulations when starting from a folded state or an unfolded state (Figures S6, S10, S8, and S12). To evaluate the sampling efficiency of the different algorithms, we computed the number of transitions between the folded and unfolded states per μs for each simulation algorithm. Chignolin and Trp-Cage were considered folded when the RMSD of the backbone atoms to the 5awl and 1l2y structures, respectively, was below 0.2 nm (as in ref 18) and unfolded when the RMSD was above 0.25 nm.

The number of fold–unfold transitions per μs for CLN025 is shown in Figure 4A. SST2 and REST2 simulations were on the same order (about 25 transitions per computed μs), with a slight advantage for REST2 simulations, as ST and SST1 simulations showed slightly fewer transitions (about 15∓20 transitions per computed μs).

**Figure 4:**
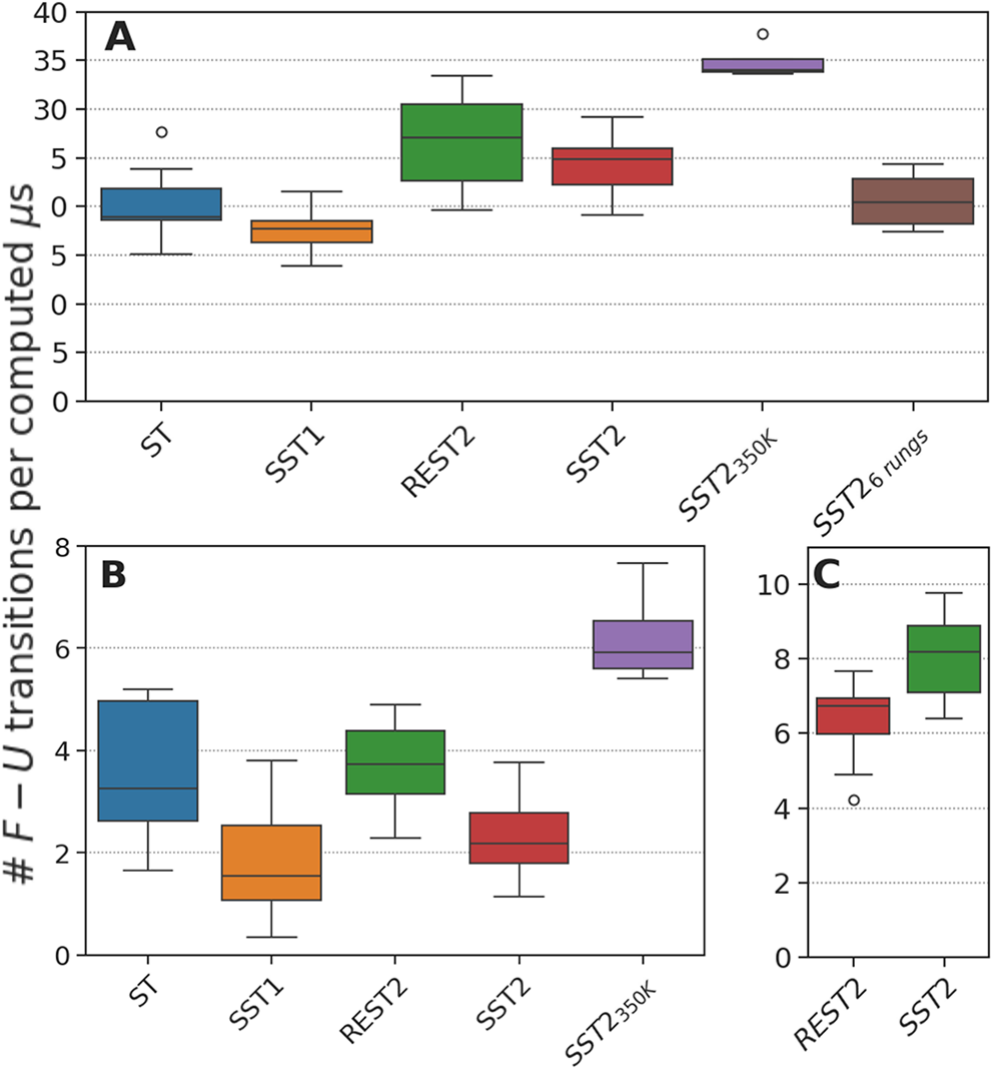
Fold/unfold or bond/unbound transitions. Number of fold/unfold transitions per μs for CLN025 (A), Trp-Cage simulations (B), and the number of bond/unbound transitions for the p97/PNGase complex (C). Simulation frames with the proline ω angle in the *cis* conformation were excluded to compute the fraction of folded conformations. Boxes represent the quartiles, while the whiskers show the rest of the distribution computed over 4 to 8 replicas. The ST, SST1, REST2, SST2, *S*ST2_350K_, and SST2_6 rungs_ simulations are represented by blue, orange, green, red, violet, and brown bars, respectively.

To understand the effect of the reference temperature *T_ref_* on the sampling efficiency, we performed SST2 simulations of CLN025 starting from a folded state with *T_ref_* = 350 *K* (*F*_350 K_). As shown in Figure 4A, the number of transitions per μs was significantly larger than that of all other simulations, averaging about 35 transitions per computed μs.

For Trp-Cage, the number of transitions between the folded and unfolded states seems to be an order of magnitude lower than that of CLN025 (Figure 4B). The ST, SST1, SST2, and REST2 simulations were on the same order of magnitude (about 2 to 4 transitions per computed μs). Differences were observed between, on the one hand, SST1 and SST2 simulations, with about 2 transitions per computed μs (1.8 ± 1.2 and 2.3 ± 1.0 transitions per computed μs, respectively), and, on the other hand, ST and REST2 simulations with about 4 transitions per computed μs (3.5 ± 1.3 and 3.7 ± 0.9 transitions per computed μs, respectively). Note that the number of transitions per μs was higher for the SST2 simulations than for the SST1 simulations.

On the other hand, changing the reference temperature to 350 K (*F*_350 K_) resulted in a higher number of transitions (6.2 ± 1.0 transitions per computed μs) than all other simulations. The difference between *T*_ref_ = 350 K and *T*_ref_ = 300 K was more pronounced for the Trp-Cage simulations than for the CLN025 simulations, with a difference of more than a factor of 2.

#### Melting Temperature

We computed the fraction of folded conformations as a function of temperature or λ for each simulation. For REST2 and SST2 simulations, we computed the effective temperature using eq 18 proposed by Stirnemann and Sterpone.^18^

The stability curve of CLN025, depicting the fraction of folded conformations as a function of temperature, is shown in Figure 5A. The melting temperature (*T_m_*) of CLN025 was estimated as 343 ± 4 K for *SST*2 and 335 ± 11 K for *REST*2, in close agreement with the experimental melting temperature of 343 K.^25^ For ST simulations, the melting temperature was 385 ± 13 K. REST2 and SST2 simulations accurately estimated the melting temperature of CLN025, whereas Stirnemann and Sterpone^18^ underestimated the melting temperature by 20 K with REST2, but with a different force field.

**Figure 5:**
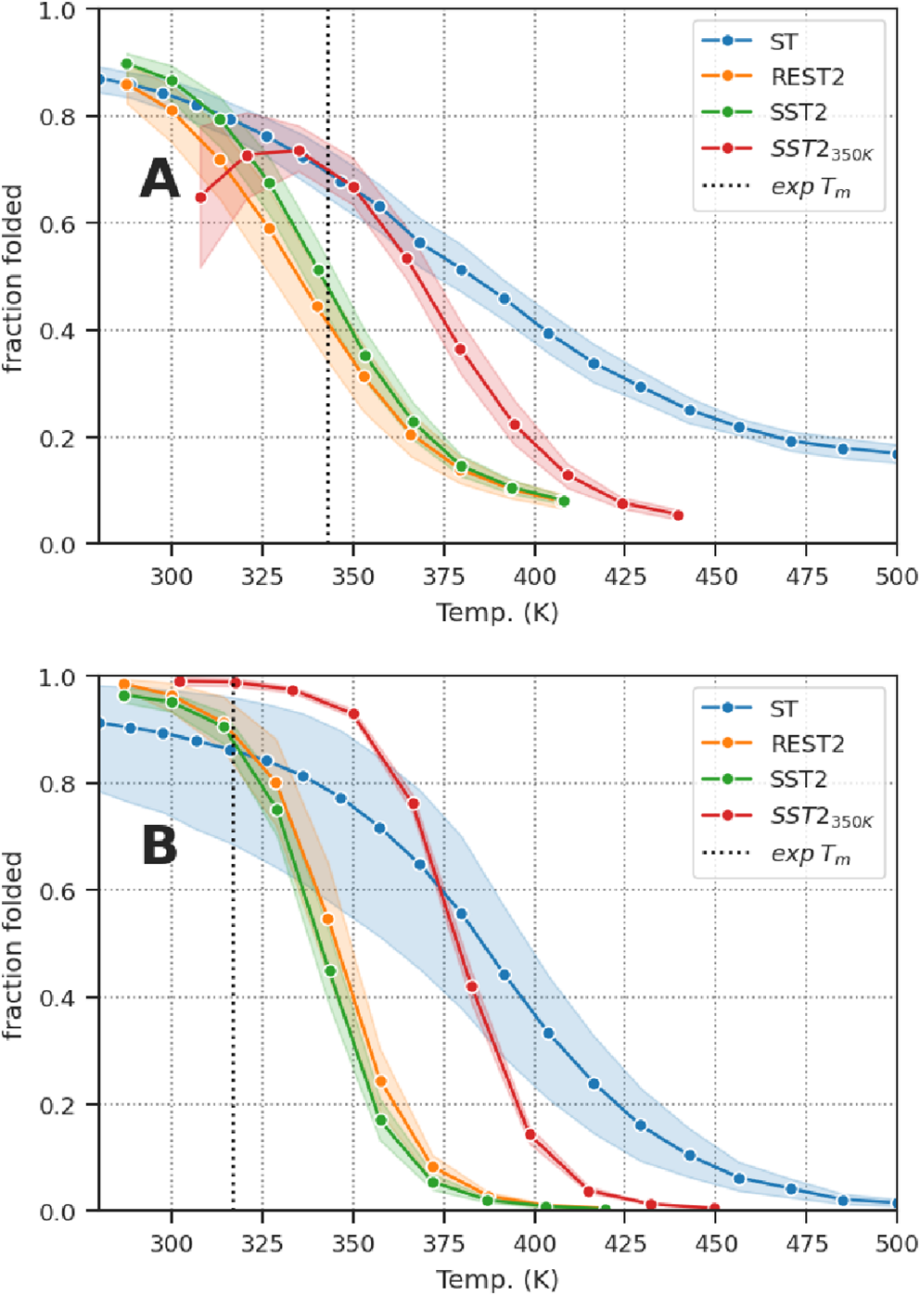
Stability curve. Fraction of folded conformation as a function of temperature for CLN025 (A) and Trp-Cage (B) simulations. Simulation frames with the proline ω angle in the *cis* conformation were excluded to compute the fraction of the folded conformation. The transparent layer around the plain line displays the 95% confidence interval computed over the 4 replicas. CLN025 and Trp-Cage were considered folded when their RMSD values were below 0.2 nm.

However, increasing the reference temperature to 350 K (SST2_350 K_) resulted in a higher melting temperature of 358 ± 11 K, overestimating the experimental value by 20 K. This overestimation was of the same order of magnitude as in the SST1 simulation (363.4 ± 17 K, where the melting temperature was computed without temperature correction as in SST2; see Figure S33).

Figure 5B shows the stability curve of Trp-Cage, which plots the fraction of the folded conformation as a function of temperature. The melting temperature (*T*_m_) of Trp-Cage was estimated to be 341 ± 5 and 346 ± 4 K for SST2 and REST2, respectively, with a ∼20 K overestimation of the experimental melting temperature of 317 K.^27^

For SST2_350 K_, the overestimation was even more pronounced at 379 ± 1 K. This overestimation was of the same order of magnitude as that in the ST simulations (378 ± 42 K), as SST1 simulations showed a reduced overestimation with a melting temperature of 358 ± 23 K (see Figure S34 for details).

Compared to Stirnemann’s REST2 simulations, which predict a melting temperature of about 320 K,^18^ our simulations overestimate the melting temperature by 20 K. In Stirnemann’s REST2 simulations, the force field used was Amberff99SB,^26^ while in our simulations, we used Amberff14SB.^32^ This difference in the force field could explain the difference in the melting temperature for CLN025 and Trp-Cage, especially as the force field has been shown to have a strong influence on the stability of Trp-Cage.^27^ This suggests that the ∼20 K overestimation of Trp-Cage melting temperature likely reflects limitations of the force field rather than shortcomings of the SST2 sampling scheme.

A large error bar was observed for the Trp-Cage *ST* simulation. The main reason seems to be that during the *F*_*ST*_(4) simulation, the protein was trapped in an unfolded state for more than 82% of the simulation time. Proline 18 was in the *cis* conformation for more than 32 μs. As we excluded frames with proline *cis* conformation, the melting curve in the simulation *F*_*ST*_(4) was computed for only ∼7 μs of the simulation time. During the first 33 μs of the *F*_*ST*_(4) simulation, the protein reached the folded state in only 3.6% of the simulation time, whereas for all ST simulations, the folded state was reached in 61.1% of the simulation time. This low frequency of the folded state may have strongly affected the weight estimation of the *F*_*ST*_(4) simulation and consequently biased the stability curve as a function of temperature. This might explain the high divergence from the other simulations (Figure S33).

Our results show that SST2 simulations provide an efficient alternative to ST simulations for sampling protein folding as a function of temperature, achieving a comparable sampling efficiency with fewer rungs. In the two *toy systems*, SST2 simulations show more transitions per μs than the SST1 simulations. The comparison of the REST2 and SST2 simulations was different for the two systems. For chignolin, the number of transitions per computed *μs* was similar for the SST2 and REST2 simulations, but for Trp-Cage, the number of transitions was more than 2-fold lower for SST2 compared to REST2 simulations.

The correlation between the RMSD and λ values provides insights into the conformational dynamics of the studied proteins. The accurate estimation of melting temperatures suggests the applicability of SST2, as REST2 simulations for studying protein folding as a function of temperature at a moderate computational cost (on the order of 500 GPU hours, using a nonprofessional GPU card, with 2 CPUs), but with an easier computation implementation for SST2 than REST2. SST2 is as easy to launch a standard OpenMM simulation, as REST2 parallelization can be more complex to implement efficiently.

### p97/PNGase Complex

For a protein–peptide complex, SST2 and REST2 ensured effective sampling of binding and unbinding events (Figure S24, S25 and 6C), with an average of 8.0 ± 1.1 and 6.3 ± 3.9 binding events per computed *μs* for SST2 and REST2, respectively (the peptide was considered bound if its backbone RMSD to 2hpl was less than 0.5 *nm*, as it was considered unbound if its RMSD was greater than 1.0 nm). However, it was observed that in several cases, the peptide was located in the binding site in a conformation that was not considered to be bound (with RMSD to the bound conformation 2hpl^31^ between 0.5 nm and 1.0 nm).

**Figure 6:**
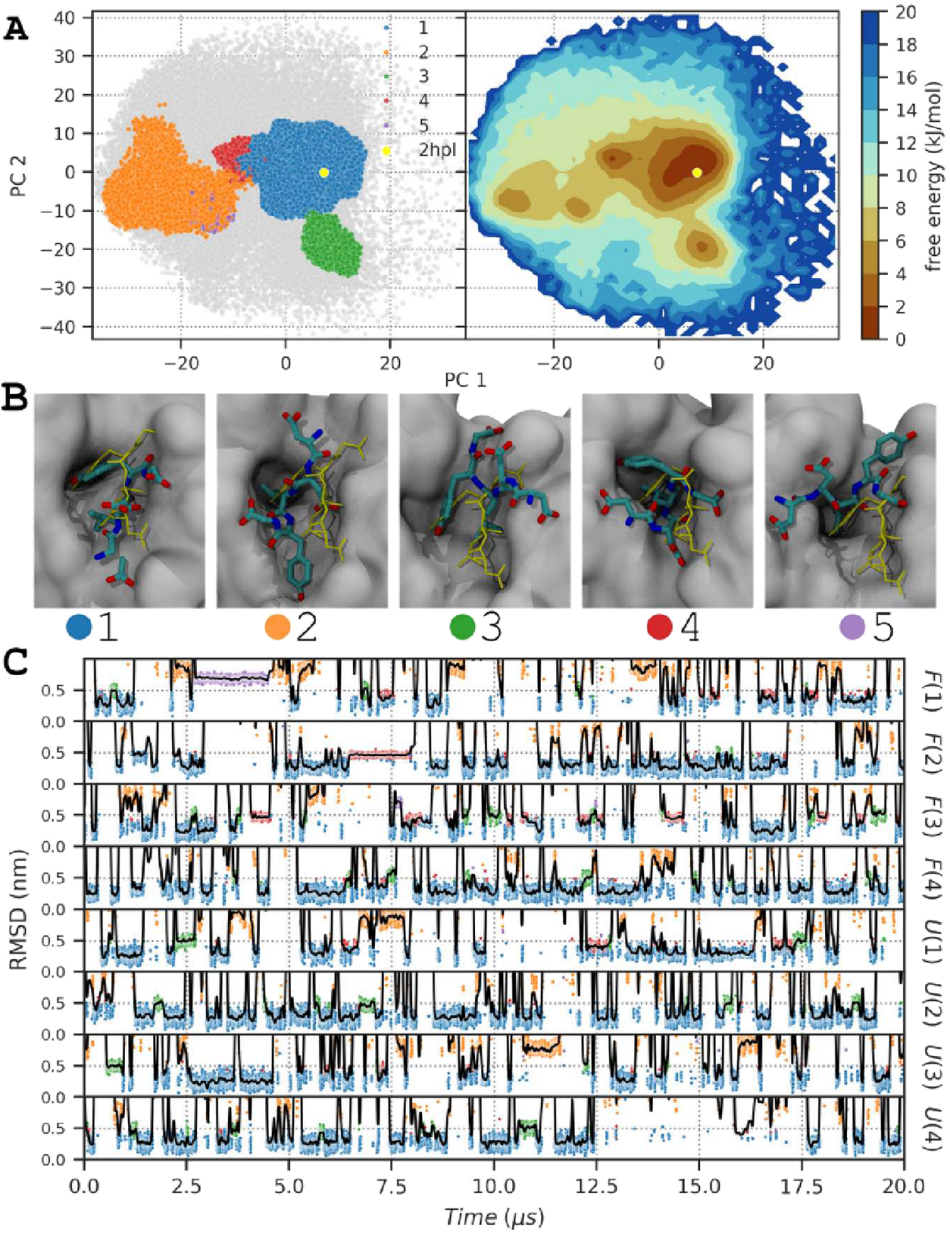
Peptide conformation in the binding site during the SST2 simulation. (A) Projection of the first two principal components of p97 backbone atoms computed after trajectory alignment on PNGase backbone atoms. Only simulation frames where the peptide is in the binding site are displayed (peptide RMSD lower than 1.0 nm). The left panel displays the projection of the clustered simulation frames colored by clusters, and the nonclustered frames are displayed as gray points. The right panel displays the free energy landscape computed from the projection of the binding site simulation frames. The reference structure 2hpl^31^ conformation is represented by a yellow dot. (B) Snapshot of the five cluster structures of the p97 peptide in the binding site of PNGase. The structure closest to the average structure of each cluster is displayed. PNGase is displayed as a gray surface, and the peptide heavy atoms are displayed as licorice colors by atom type. The peptide reference bound conformation (2hpl) is displayed as a yellow licorice. (C) RMSD as a function of time for the p97 peptide. RMSD was computed on the backbone atoms of the p97 peptide after structural alignment of the PNGase backbone atoms to the reference structure 2hpl. Raw RMSD values are displayed as dots, colored as a function of clusters: blue, orange, green, red, and purple for clusters 1, 2, 3, 4, and 5, respectively. A smooth Gaussian filter with σ = 20 applied to the RMSD values is displayed as a black line. For clarity, only RMSD values between 0 and 1.0 *nm* are displayed, and the complete RMSD curve is shown in the Supporting Information (Figure S24).

We computed the PCA on the SST2 and REST2 aggregated simulations on the peptide backbone atom coordinates and used the first four components to cluster the binding sites using HDBSCAN. Analyzing all the aggregated data (SST2 and REST2 simulations), the largest cluster is the closest to the bound conformation, with an average RMSD of 0.44 ± 0.32 nm to 2hpl and contains the largest fraction of the simulation frames (63.5%), while the second largest cluster contains only 3.0% of the simulation frames. The largest cluster consisted of 97% of the simulation frames, where the peptide RMSD to 2hpl was below 1 nm. We later used a 1 nm RMSD cutoff to define the peptide conformations in the binding site.

We then analyzed in detail the peptide conformations in the binding site using PCA limited to this subset of simulation frames, followed by HBSCAN clustering. The clustering results revealed five distinct conformations of the peptide in complex with PNGase (Figure 6). Cluster 1 is the most populated conformation, encompassing 36.7 ± 10.0% of the simulation frames. With an average RMSD of 0.26 ± 0.07 nm to 2hpl, this cluster is the closest to the bound conformation. Clusters 2 and 5 account for 3.3 ± 3.1% and 0.6 ± 2.2% of the frames, respectively, and have an average RMSD of 0.83 ± 0.08 and 0.69 ± 0.04 nm, respectively, suggesting conformations further away from the bound state. Clusters 3 and 4, comprising 2.2 ± 1.5% and 1.6 ± 2.7% of the frames, respectively, represent intermediate states with an average RMSD of 0.49 ± 0.06 and 0.45 ± 0.03 nm, respectively.

Overall, during SST2 and REST2 simulations, the peptide explored the same conformational space (Figures 6A and S26A); however, SST2 simulations explored the alternative conformations of the peptide in the binding site more than REST2 simulations. The occupancy of cluster 1 was 30.7 ± 10.1% and 42.8 ± 5.2% for SST2 and REST2 simulations, respectively, because alternative conformations were more populated in SST2 simulations (4.7 ± 3.7, 2.9 ± 1.5, 2.9 ± 3.5, and 1.2 ± 3.1% for clusters 2, 3, 4 and 5, respectively) than in REST2 simulations (1.8 ± 1.3, 1.5 ± 1.2, 0.3 ± 0.3, and 0.03 ± 0.07% for clusters 2, 3, 4, and 5, respectively). Importantly, while these additional clusters are not represented in the experimental structure, they are physically plausible intermediates that highlight the ability of SST2 to sample beyond the single bound conformation captured in the crystal structure.

In the simulation *F*(1), the peptide remained in the cluster 2 and 5 conformations for extended periods, for 11.1 and 8.8% of the simulation time, respectively, as the residence time of cluster 1 was reduced to 17.4% (Figure 6).

Figure 7 shows the fraction of peptide bound as a function of temperature for the SST2 and REST2 simulations. Similar to the fraction of folded peptide in the CLN025 and Trp-Cage simulations, the fraction of the bound peptide shows a clear dependence on temperature. The curves for the REST2 and SST2 simulations are similar, but the error for the *SST*2 simulations is more pronounced, mainly due to the *F*(1) simulation.

**Figure 7:**
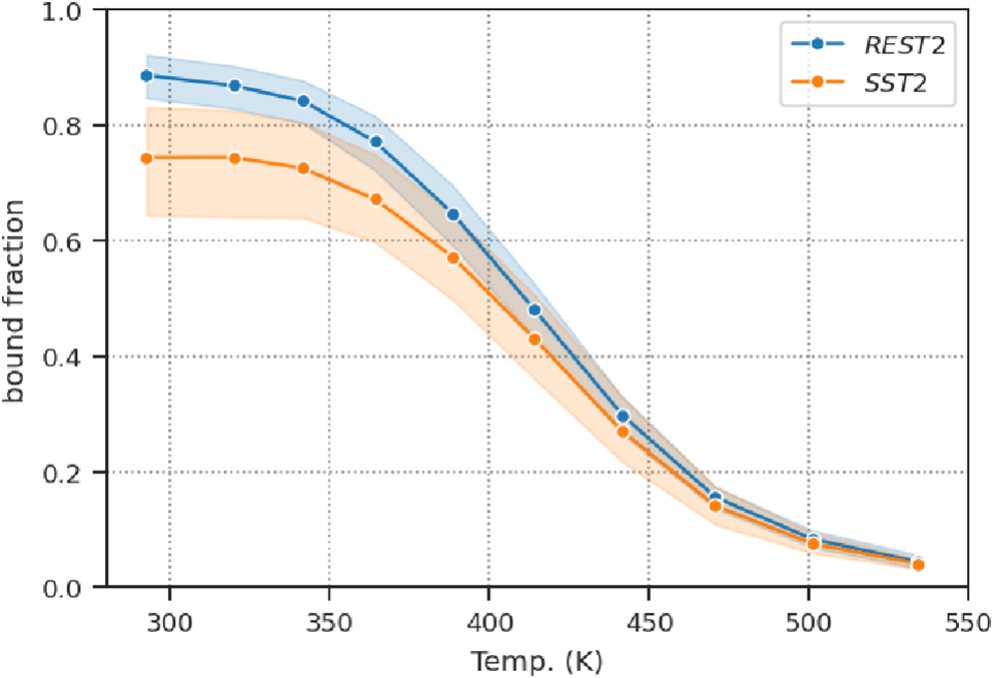
Peptide binding stability. The fraction of the peptide bound as a function of temperature. A peptide was considered bound when the peptide backbone RMSD to the reference structure 2hpl^31^ was less than 0.5 nm. The transparent layer around the plain line displays the 95% confidence interval computed over the 4 replicas.

During simulation *F*(1), the prolonged residence time in intermediate conformations 2 and 5 had a marked effect on the fraction of the peptide bound as a function of temperature, as these two conformations were not classified as bound. This explains the substantial error bars observed in the fraction of the peptide bound as a function of temperature for the *SST*2 simulation group (Figures 7 and S35). A more detailed analysis of these conformations, including free energy maps, receptor flexibility, and the influence of loops 55–59 on ligand binding, is provided in the Supporting Information.

### Simulation Convergence

As shown in previous studies,^22,28,33,34^ accurate weight estimation is crucial for efficient ST simulations. The convergence of the weight estimation in the SST1, SST2, and ST simulations was evaluated for all systems. The results are presented in the Supporting Information and demonstrate that the SST1, SST2, and ST simulations exhibit comparable convergence patterns, with weight estimation reaching stability after 2–10 μs depending on the system. The folded or bound fraction curves as a function of temperature converge at a faster rate than that of the weight estimation.

Eventually, the SST2 computing performance was evaluated, and the computing performance was estimated to be about 20% slower compared to that of a conventional MD simulation (see Supporting Information).

## Discussion and Conclusion

The Simulated Solvent Tempering 2 (SST2) algorithm presented in this study represents a novel approach to improve the efficiency of MD simulations, particularly in the context of exploring the conformational space and thermal stability of biomolecular systems.

Our comparisons showed that SST2 achieves a performance close to that of ST and REST2 in terms of conformational space sampling; as in the two *toy systems*, SST2 was slightly more efficient than SST1.

We have shown that SST2 simulations can achieve comparable rung transition probabilities with less than a third of the rungs required for the ST simulations and that with the same number of rungs, SST2 displayed a factor 2–3 higher probability of transition than REST2 simulations. One must also keep in mind that for REST2 or SST2, the number of rungs depends only on the size of the solute; it is then possible to achieve the same efficiency as ST, but for larger and more complex systems. In addition, the adjustment of the reference temperature in SST2 demonstrated its potential to exceed the efficiency of the ST.

One objective of our study was to assess whether SST2, similar to REST2, could accurately reconstruct the thermal stability curves of the simulated peptides. Our simulations of chignolin CLN025 and Trp-Cage showed that SST2 does indeed reproduce the thermal stability curves with fidelity comparable to that of REST2. It should be noted that increasing the reference temperature to 350 K resulted in an overestimation of the melting temperature. In fact, it is expected, as SST2 and ST simulations are consistent when λ = 1.0 for SST2 and when *T* = *T_ref_* for ST since they have identical Hamiltonians at these reference points. Therefore, increasing the reference temperature *T_ref_* to 350 K logically shifts the entire temperature scale upward, resulting in a corresponding overestimation of *T_m_*. This behavior, which also applies to REST2, is not due to errors in the mean-field treatment but is a direct consequence of the choice of *T_ref_*. The mean-field approximation used to estimate the effective temperature (eq 18) has inherent limitations. However, it is particularly useful in comparative studies, where absolute accuracy is less critical than the relative behaviors of different systems or methods. As in Timr and Sterpone,^35^ where the authors were able to use REST2 to study the stability of the chymotrypsin inhibitor in complex environments (two different protein crowded environments), and showed the effect of the solvent on the stability of the protein. The demonstrated ability of SST2 to probe interactions and conformational dynamics in such complex environments suggests that it would be well-suited for this type of investigation. In addition to being limited to the study of complex environments, ST and REMD are also constrained by current force fields, which are known to overstabilize native-like protein structures, a well-documented issue that can lead to systematic overestimation of melting temperatures across methods.

The major weakness of SST2 compared to REST2 is the weight estimation, similar to ST compared to REMD. Stirnemann and Sterpone^18^ have shown that REST2, with 12 replicas, converges on a 150 and 250 ns time scale (corresponding to simulated times of 1.8 and 3 μs) for the CLN025 and Trp-Cage stability curves, respectively. In our study, the SST2 simulations required 0.5–1 and 2–10 μs to reach convergence for CLN025 and Trp-Cage stability curves, respectively. A comparison with our REST2 simulations shows a similar convergence for the equivalent computed simulation time. SST2 achieves convergence comparable to REST2 in terms of total computation time, while also offering advantages in terms of ease of implementation. While REST2 benefits from faster convergence per unit time due to parallelism, SST2 can offer similar efficiency in terms of the total computational cost with significantly reduced technical complexity. Moreover, if a better weight estimation method is found, SST2 would theoretically be more efficient than REST2. A potential strategy to tackle this challenge is the use of machine learning-based approaches for weight estimation, which, to our knowledge, have not yet been applied to simulated tempering but could accelerate the convergence and further improve the efficiency of SST2.

Another practical difference between SST2 and REST2 is that SST2 has a continuous simulation trajectory that starts with a single initial configuration. Although only the configurations at λ = 1.0 correspond directly to the native physical system, the uninterrupted trajectory enables a more natural dynamical evolution between exchange attempts, even when intermediate λ values deviate from the physical reality. This continuous trajectory is particularly beneficial for capturing slow conformational or environmental relaxation processes, such as solvent rearrangements or membrane reorganization, where large-scale changes require sustained sampling rather than fragmented simulation intervals. While caution is warranted when interpreting dynamics at λ ≠ 1.0, the ability to track the system’s evolution across a consistent trajectory may provide insight into the slow processes influencing sampling at λ = 1.0.

For example, in the p97/PNGase complex, the RMSD of the receptor backbone over the full simulation time (0–20 and 0–1 μs) was slightly higher in SST2 (1.52 ± 0.40 Å) than in REST2 (1.32 ± 0.30 Å, see Figure S29), probably reflecting the broader exploration in SST2. However, when comparing only the first microsecond (0–1 μs), both methods yielded similar receptor RMSD values (1.38 ± 0.28 Å for SST2 and 1.32 ± 0.30 Å for REST2). This suggests that SST2 may offer an advantage for observing slow-relaxing structural features, particularly over extended simulation times.

The accurate estimation of weights in both SST2 and ST simulations remains a significant challenge. As shown in our simulations, the weight estimation process can be affected by slow conformational transitions and could reach convergence only after ∼10 μs for the case of Trp-Cage. The utilization of techniques such as the Weighted Histogram Analysis Method (WHAM)^23^ has the potential to enhance the precision of weight determination, thereby addressing the inherent complexities associated with achieving reliable weight values. Nevertheless, in order to accurately estimate the weights, it is necessary to sample the folded and unfolded forms of the protein at a minimum frequency at all temperatures or λ. This presents a challenge for proteins that fold slowly, as the simulation time must be sufficiently long to ensure adequate sampling of the conformational space.

As shown in Figures S20 and S21, the lambda rung occupation is not uniform, with low-temperature rungs being more populated than others in the ST and SST2 simulations. As in ref 28, skewing weights toward the higher-temperature rungs could improve the sampling efficiency.

One of the unexpected outcomes of the simulation analysis was the impact of proline isomerization on the folding process. It was unexpected that Trp-Cage could be trapped in an unfolded state for more than 32 μs due to a proline residue in the cis conformation. This observation highlights the necessity of considering slow conformational transitions in protein simulations, as they can significantly impact the accuracy of the results. This is particularly relevant, as Doshi et al. have shown that the free energy of *cis–trans* isomerization is underestimated by ∼6 kcal·mol^–1^ in the Amberff99SB force field.^36^ Several solutions have been proposed to address the issue of isomerization sampling, as in ref 37, where the authors accelerated the sampling of *N*-methylated *cis–trans* amide isomers by scaling directly the force field parameters and the ω dihedral angle torsion constants. An alternative solution is to use a broader range of temperatures for SST2; employing temperatures up to 700 K, as in ref 38, accelerates significantly the time associated with the proline *cis–trans* transition. Nevertheless, the use of elevated temperatures implicates the use of additional replicas, which could potentially increase again the computational cost of the ST and REMD simulations. Conversely, for REST2 and SST2, the number of ladders is sufficiently limited, thereby reducing the likelihood of this becoming a significant issue. The exclusion of the ω dihedral angles from the scaled intramolecular energy term in the SST2 simulations represents a significant improvement, as it allows efficient sampling of the conformational space without being hindered by slow transitions.

Similarly, experimental data could be used to exclude additional dihedral angles from the scaled intramolecular energy to guide or prevent the simulation of the conformational states that are present or not present experimentally. While SST2 is an efficient method, it is limited by the folding or binding time of the object under study. With similar concepts, derivatives of REST2 methods, such as generalized REST (gREST)^39^ restrain the scaled energy terms only to the dihedral angles and/or the CMAP terms of the solute. This enables a further reduction in the number of λ rungs, thereby reducing the computational cost and improving temperature sampling. This method could be adapted and implemented effortlessly in SST2 with the objective of enhancing the sampling efficiency of slow-folding proteins. A hybrid method combining REST2 and temperature REMD, Replica Exchange with Hybrid Tempering (REHT),^40^ has shown improvement in conformational space sampling and could be adapted to methodologies that use a single simulation, such as SST2.

Another promising direction is the integration of machine learning-based postprocessing approaches, such as Denoising Diffusion Probabilistic Models (DDPMs), which have recently been adapted for REMD^41^ and REST2.^42^ Since SST2 also relies on Hamiltonian scaling across modified potential energy surfaces, this concept is directly applicable. DDPMs may learn the joint distribution of solute configurations and their rescaled energies and then exploit this knowledge to refine the reconstruction of free energy landscapes. Such approaches may help resolve subtle barriers that remain undersampled during the simulation itself and extend the predictive power of SST2 toward long-time scale processes without additional raw sampling.

The successful application of SST2 to chignolin CLN025 and Trp-Cage paves the way for broader utilization in the study of diverse biomolecular systems. Note that REST2 was clearly more efficient than SST2 for Trp-Cage simulations, with a factor of 2 higher number of transitions per computed μs. However, as evidenced by the p97/PNGase complex simulations, SST2 is also capable of efficiently sampling large systems as protein–peptide complexes, with a higher sampling efficiency than REST2, thereby offering invaluable insights into the dynamics of ligand–receptor binding and recognition. SST2 shows great promise for applications beyond isolated small protein simulations. Its capacity to efficiently sample conformational space makes it a promising candidate for the study of systems involving ligands, peptides, or proteins within complex environments such as lipidic membranes or in complex with a receptor. This versatility establishes SST2 as a valuable tool for investigating biomolecular interactions and dynamics in realistic settings.

In conclusion, the SST2 algorithm, which is presented as an amalgamation of ST and REST2, demonstrates promising advancements in MD simulation sampling. Our comparative analysis and application to specific systems have highlighted the potential of SST2 to efficiently sample conformational space and reconstruct thermal stability curves. The adaptability of SST2, evidenced by its ability to surpass ST, SST1, and REST2 efficiency under specific conditions, positions it as a valuable addition to the toolkit of computational biophysicists.

## Materials and Methods

We first present the concepts and theories behind REMD, ST, REST, and REST2, and eventually, the theory and implementation of SST2.

### Classical REMD

Replica Exchange Molecular Dynamics (REMD)^9^ simulates multiple copies (or *replicas*) of a molecular system at different temperatures. Swaps between neighboring replicas are attempted at regular time intervals. The probability of an exchange between temperature *m* and *n* is calculated using a Metropolis criterion *p*_*mn*_ = min­(1, *e* ^Δ_*mn*_^*REMD*^^) with

1 $$\Delta_{\mathrm{mn}}^{\mathrm{REMD}}=\beta_{m}\left[E_{m}\left(x_{m}\right)-E_{m}\left(x_{n}\right)\right]-\beta_{n}\left[E_{n}\left(x_{m}\right)-E_{n}\left(x_{n}\right)\right]$$

where β_*m*_ = 1/(*k*_*B*_ *T*_*m*_), *E*_*m*_ and *x*_*m*_ are the potential energy and the configuration of the system at temperature *T*_*m*_, respectively. Since the potential energy is independent of the temperature, we obtain

2 $$\Delta_{\mathrm{mn}}^{\mathrm{REMD}}=\left(\beta_{m}-\beta_{n}\right)\left[E\left(x_{m}\right)-E\left(x_{n}\right)\right]$$

### REST

In the first version of Replica Exchange with Solute Tempering (REST),^12^ instead of just increasing the temperature as in REMD, the Hamiltonian is also updated as the temperature increases to keep the solvent at cold or ambient temperature (*T*_*ref*_) using

3 $$E_{m}^{\mathrm{REST}}\left(X\right)=E_{\mathrm{pp}}\left(X\right)+\frac{\beta_{\mathrm{ref}}}{\beta_{m}}E_{\mathrm{ww}}\left(X\right)+\frac{\beta_{\mathrm{ref}}+\beta_{m}}{2\beta_{m}}E_{\mathrm{pw}}\left(X\right)$$

where *X* is the configuration of the system, *E*_*pp*_ is the solute intramolecular energy, *E*_*pw*_ is the solute–solvent interaction energy, and *E*_*ww*_ is the solvent intramolecular energy.

Although REST has been shown to accelerate the convergence of the conformational space distribution for small molecules, it has proven less effective than temperature REMD for larger systems such as Trp-Cage and β hairpin.^13^ To overcome this limitation, several improvements to REST have been implemented.

Hamiltonian modifications inspired by Moors et al.^43^ and Terakawa et al.^44^ led to an improved version of REST called REST2. In REST2 ^14^ simulations, the replicas are all run at the same temperature *T_ref_*, but the replicas evolve with different Hamiltonians. For replica *m* running at λ_*m*_ = β_*m*_/β_*ref*_ = *T*_*ref*_/*T*_*m*_ (considered as an approximation of *T*_*m*_)­

4 $$E_{m}^{\mathrm{REST}2}\left(X\right)=\frac{\beta_{m}}{\beta_{\mathrm{ref}}}E_{\mathrm{pp}}\left(X\right)+\sqrt{\frac{\beta_{m}}{\beta_{\mathrm{ref}}}}E_{\mathrm{pw}}\left(X\right)+E_{\mathrm{ww}}\left(X\right)$$

or

5 $$E_{m}^{\mathrm{REST}2}\left(X\right)=\lambda_{m}E_{\mathrm{pp}}\left(X\right)+\sqrt{\lambda_{m}}E_{\mathrm{pw}}\left(X\right)+E_{\mathrm{ww}}\left(X\right)$$

The acceptance ratio for the exchange between replicas *m* and *n* is defined by

6 $$\Delta_{\mathrm{mn}}^{\mathrm{REST}2}=\left(\beta_{m}-\beta_{n}\right)\left[\left(E_{\mathrm{pp}}\left(X_{n}\right)-E_{\mathrm{pp}}\left(X_{m}\right)\right)+\frac{\sqrt{\beta_{\mathrm{ref}}}}{\sqrt{\beta_{m}}+\sqrt{\beta_{n}}}\left(E_{\mathrm{pw}}\left(X_{n}\right)-E_{\mathrm{pw}}\left(X_{m}\right)\right)\right]$$

Since solute–solute interactions are scaled equally compared to solvent–solvent interactions by β_*m*_/β_*ref*_ in REST and REST2, the difference comes from the solute–solvent interaction, which is scaled by (β_*m*_ + β_*ref*_)/2β_*m*_ in REST and $\sqrt{\beta_{m}/\beta_{\mathrm{ref}}}$ in REST2. REST2 by using weaker solute–solvent interactions at high temperatures, favors the sampling of folded conformations even at high temperatures.

### Simulated Tempering

In simulated tempering (ST),^10,11^ a single copy of the system is simulated and the temperature is periodically updated between a predetermined set of values. As in REMD, the Metropolis algorithm is used to decide whether or not to accept a temperature swap; one of the main advantages over REMD is that the temperature swap depends on only one simulated system rather than two, which speeds up the temperature transition. Zhang and Ma^21^ have shown that ST gives a higher rate of traversal of energy space with the reserve of an accurate partition function (ST weights *w*_0_, *w*_1_, ..., *w*_*i*–1_).

The acceptance rate is given by *p*_*mn*_ = min­(1, *e* ^Δ_*mn*_^*ST*^^) with

7 $$\Delta_{\mathrm{mn}}^{\mathrm{ST}}=\left(\beta_{m}E_{m}\left(X\right)-w_{m}\right)-\left(\beta_{n}E_{n}\left(X\right)-w_{n}\right)$$

The potential energy function *E*_*m*_ (*X*) is independent of the temperature *T*_*m*_, so *E*_*m*_ = *E*_*n*_, which gives

8 $$\Delta_{\mathrm{mn}}^{\mathrm{ST}}=\left(\beta_{m}-\beta_{n}\right)E+\left(w_{n}-w_{m}\right)$$

In ST simulations, the correct calculation of the exact partition function is particularly important for efficient ST computation. To compute the weights, we used the method proposed by Park and Pande^34^

9 $$w_{m+1}=w_{m}+\left(\beta_{m+1}-\beta_{m}\right)\frac{\left\langle E_{m+1}\right\rangle +\left\langle E_{m}\right\rangle }{2}$$

Since *E*_*m*_ must be calculated beforehand, we used the *on the fly* weight calculation developed by Nguyen et al.,^33^ which allows to approximate *w*_*m*+1_ if no simulation has been calculated at *T*_*m*+1_

10 $$w_{m+1}=w_{m}+\left(\beta_{m+1}-\beta_{m}\right)\left\langle E_{m}\right\rangle$$

### Simulated Solute Tempering (SST1)

In the SST implementation of REST in ST,^23^ the authors modified the potential energy function using

11 $$E_{m}^{\mathrm{REST}}\left(X\right)=E_{\mathrm{pp}}\left(X\right)+\sqrt{\frac{\beta_{\mathrm{ref}}}{\beta_{m}}}E_{\mathrm{pw}}\left(X\right)+\frac{\beta_{\mathrm{ref}}}{\beta_{m}}E_{\mathrm{ww}}\left(X\right)$$

Using eq 11 in ([Disp-formula eq8]) gives

12 $$\Delta_{\mathrm{mn}}^{\mathrm{SST}1}=\left(\beta_{m}-\beta_{n}\right)E_{\mathrm{pp}}+\left(\sqrt{\beta_{\mathrm{ref}}\beta_{m}}-\sqrt{\beta_{\mathrm{ref}}\beta_{n}}\right)E_{\mathrm{pw}}-\left(w_{m}-w_{n}\right)$$

While SST1 combines the advantages of ST and REST, it suffers from the same limitations as REST and has not been adopted by the community as a reference method for protein folding.

### Simulated Solute Tempering 2 (SST2)

We used a slightly modified version of the original implementation of REST2. In the approach proposed in ref 18, only the proper torsion terms of the solute were scaled among the bonded terms

13 $$E_{m}^{\mathrm{SST}2}\left(X\right)=\frac{\beta_{m}}{\beta_{\mathrm{ref}}}E_{\mathrm{pp}}^{\left(1\right)}\left(X\right)+E_{\mathrm{pp}}^{\left(2\right)}\left(X\right)+\sqrt{\frac{\beta_{m}}{\beta_{\mathrm{ref}}}}E_{\mathrm{pw}}\left(X\right)+E_{\mathrm{ww}}\left(X\right)$$

or using λ_*m*_ = β_*m*_/β_*ref*_

14 $$E_{m}^{\mathrm{SST}2}\left(X\right)=\lambda_{m}E_{\mathrm{pp}}^{\left(1\right)}\left(X\right)+E_{\mathrm{pp}}^{\left(2\right)}\left(X\right)+\sqrt{\lambda_{m}}E_{\mathrm{pw}}\left(X\right)+E_{\mathrm{ww}}\left(X\right)$$

where *E*_*pp*_^(1)^ is the scaled solute intramolecular energy (LJ, Coulomb, and proper torsions), *E*_*pp*_^(2)^ is the unscaled solute intramolecular energy (bonds, angles, and improper torsions), *E*_*pw*_ is the solute–solvent interaction energy, and *E*_*ww*_ is the solvent intramolecular energy.

The acceptance ratio is given by *p*_*mn*_ = min­(1, *e* ^Δ_*mn*_^*SST2*^^), where inserting ([Disp-formula eq13]) into ([Disp-formula eq8]) gives

15 $$\Delta_{\mathrm{mn}}^{\mathrm{SST}2}=\left(\beta_{m}-\beta_{n}\right)\left[E_{\mathrm{pp}}^{\left(1\right)}\left(X\right)+\frac{\sqrt{\beta_{\mathrm{ref}}}}{\sqrt{\beta_{m}}+\sqrt{\beta_{n}}}E_{\mathrm{pw}}\left(X\right)\right]+\left(w_{n}-w_{m}\right)$$

This equation is equivalent to the original SST method ([Disp-formula eq12]).

Using Δ_*mn*_^*typ*^ = Δ_*nm*_^*typ*^, we obtain

16 $$\left(w_{n}-w_{m}\right)=\left(\beta_{n}-\beta_{m}\right)\frac{\left(\langle E_{\mathrm{pp}}^{\left(1\right)}\rangle_{m}-\langle E_{\mathrm{pp}}^{\left(1\right)}\rangle_{n}\right)}{2}+\left(\sqrt{\beta_{\mathrm{ref}}\beta_{n}}-\sqrt{\beta_{\mathrm{ref}}\beta_{m}}\right)\frac{\left(\langle E_{\mathrm{pw}}\rangle_{m}-\langle E_{\mathrm{pw}}\rangle_{n}\right)}{2}$$

The exchange with neighboring *m* + 1 replicas is determined by the fluctuation of $E_{\mathrm{pp}}+\sqrt{\beta_{\mathrm{ref}}}/\left(\sqrt{\beta_{m}}+\sqrt{\beta_{m+1}}\right)E_{\mathrm{pw}}$ or, for simplicity, since β_*m*_ and β_*m*+1_ are close, we will later monitor $E_{\mathrm{pp}}+0.5\sqrt{\beta_{\mathrm{ref}}/\beta_{m}}E_{\mathrm{pw}}$ .

### Temperature Distribution

*m* temperatures have been chosen to be exponentially spaced between the two extremes *T*_min_ = *T*_0_ and *T*_max_ = *T*_*m*–1_. With *k* included in [0, 1, ..., *m* – 1], the temperatures have been computed as follows

$$T_{k}=T_{\min}{\left(\frac{T_{\max}}{T_{\min}}\right)}^{k/(m-1)}$$ 17

If *T*_*ref*_ is different from *T*_min_, then we use the same formula to compute the effective temperatures between *T*_min_ and *T*_*ref*_ and between *T*_*ref*_ and *T*_max_.

### Effective Temperature Estimation

To estimate the corrected SST2 solute temperature, we use the formula proposed by Stirnemann and Sterpone in ref 18

18 $$\left\langle \beta_{m}^{\prime }\right\rangle =\beta_{m}\left(1+\left(\sqrt{\frac{\beta_{\mathrm{ref}}}{\beta_{m}}}-1\right)\left\langle \frac{E_{\mathrm{pw}}\left(X\right)}{E_{\mathrm{pp}}\left(X\right)+E_{\mathrm{pw}}\left(X\right)}\right\rangle \right)$$

### SST2 Implementation

The ST, SST1, and SST2 protocols have been implemented using Python scripts and OpenMM.^45^ ST’s original OpenMM script, written by Peter Eastman, was modified to implement the weight calculation of Park and Pande^34^ and the *on the fly* weight calculation from Nguyen et al.^33^ The same script was also used to write the SST2 scripts.

The scaling of nonbonded interactions was done by scaling the ϵ parameters of the Lennard-Jones potential by λ_*m*_ = β_*m*_/β_*ref*_, while the charges of solute atoms were scaled by $\sqrt{\lambda_{m}}$ . The solute intramolecular energies (bonds, angles, and improper torsions) were left unchanged; for the selected proper torsion terms, the dihedral constant term *k* was scaled by λ_*m*_.

In order to accurately compute the different energy terms *E*_*pp*_, *E*_*pw*_, and *E*_*ww*_, two additional molecular systems were created in addition to the simulated systems, consisting of the solute atoms only and the solvent atoms only. At each step where the energy terms had to be computed, the additional systems were assigned the same coordinates as the simulated systems. This allows the long-term electrostatic contributions of *E*_*pp*_ and *E*_*ww*_ to be accurately computed and the nonbonded interaction contribution *E*_*pw*_ to be derived.

As in classical MD simulations, it is generally recommended to neutralize the full system when Ewald summation methods are used for long-range electrostatics. However, when the solute is charged, the solute-only and solvent-only subsystems are not neutral, which could, in principle, lead to artifacts in the energy calculation. To address this, it is important to note that the PME implicitly introduces a uniform neutralizing background charge. OpenMM versions prior to version 8.3.1 did not include the explicit correction term for this background, whereas, from version 8.3.1 onward, the correction has been implemented, ensuring consistent energy reporting in charged systems. We verified that this correction has no effect on the forces and only a very minor effect on the reported energies. Specifically, we performed 10 ns simulations of CLN025, TrpCage, and PNGase, and compared exchange acceptance with and without correction. In all three cases, ∼99.9% of the exchanges were identical (i.e., only ∼ 5 out of 5000 attempts differed). Thus, while the impact is limited to reported energies and exchange calculations, we recommend that users employ OpenMM ≥ 8.3.1 to ensure robust and consistent treatment of charged systems.

The ST, SST1, and SST2 implementations are available at github.com/samuelmurail/SST2.

To use our simulation script, we recommend using the Remd Temperature Generator Web server^46^ at https://virtualchemistry.org/remd-temperature-generator/ to calculate the SST1, SST2, or ST temperature list, or simply the number of ladders. The user should divide the expected probability by 2 (ratio of expected probability exchange between REMD and ST or REST2 and SST2). For SST2, enter the number of solute atoms for the *Number of protein atoms*, and 0 for the *Number of water molecules*.

### System Preparation

Two types of systems were prepared: those starting from an unfolded state and those starting from a folded state.

The unfolded structures were generated using the pdb_manip_py library (pdb-manip-py.readthedocs.io) and later the pdb_numpy library (pdb-numpy.readthedocs.io) to generate a linear structure of the peptide. Structure preparation and protonation were performed by using the pdbfixer module of the OpenMM package. The protocol for preparing the folded structure was similar to that for the unfolded structure, except that the implicit simulation step was skipped, as we started directly from the PDB structures 5AWL^29^ and 1L2Y^30^ for the chignolin, and Trp-Cage proteins, respectively.

The simulation of the p97/PNGase complex was prepared using the PDB structure 2HPL^31^ and the same protocol as for the folded structure. For the unfolded structure, the PNGase structure was prepared using the PDB structure 2HPJ,^31^ as the p97 peptide was prepared as the linear peptide and placed randomly around the PNGase. The same minimization, equilibration, and simulation protocols was used.

### Molecular Dynamics Simulation Preparation

The simulations were computed using the OpenMM 7.7 package.^45^ In order to circumvent the utilization of an excessively large simulation box, the chignolin and Trp-Cage linear structures were minimized using the L-BFGS algorithm for up to 10.000 steps and then submitted to an implicit solvent equilibration of 10 ns using the Langevin integrator and the GBSA-OBC solvation model^47^ with the Amber99sbNMR^48^ force field. We originally planned to use Amber14SB for implicit solvent simulations, but the implicit solvent model was not implemented in OpenMM at the beginning of the study. The obtained structures, folded structures, and protein–peptide complexes were then used as starting points for the explicit solvent simulations. The force field was Amber14SB,^32^ and proteins were solvated in a truncated octahedron box with a padding of 1.5 nm and the TIP3P water model.^49^ Where necessary, counterions were added to counteract the charge of the protein. The systems were again minimized up to 10.000 steps and equilibrated for 10 ns, and the temperature and pressure were equilibrated using a Monte Carlo barostat at a temperature of 300 K and a pressure of 1 atm. A nonbonded cutoff of 1.0 nm was used with the Particle Mesh Ewald algorithm for long-range electrostatic interactions. We used a 4 fs integration time step using heavy hydrogen assigned a mass of 3 atomic mass units.^50^

### Simulation Run

We performed ST, SST1, and SST2 simulations of CLN025 and Trp-Cage (Table 1) in an explicit solvent, starting from the unfolded and folded states (*U* and *F*). Temperature updates were performed every 2 ps for the ST, SST1, and SST2 simulations, following the protocol in ref 28, as we used friction coefficients of 10, 1, and 1 ps^–1^ for the Langevin integrator, for the ST, SST1, and SST2 simulations, respectively (see the Supporting Information and Figure S37 for a detailed discussion of the effect of the friction coefficient on the ST and SST2 simulations). For both the ST and SST2 simulations, four replicas for the folded and unfolded structures were computed for 10 and 40 μs, for CLN025 and Trp-Cage, respectively. As for the SST1 simulations, we used simulation times of 10 and 20 μs for CLN025 and Trp-Cage, respectively. The SST2 simulations used a reference temperature *T_ref_* = 300 *K*, with 10 λ rungs of 1.07, 1.00, 0.93, 0.86, 0.80, 0.75, 0.69, 0.64, 0.60, and 0.56, corresponding to solute–solute corresponding temperatures of 280.0, 300.0, 322.9, 347.5, 374.0, 402.5, 433.2, 466.2, 501.7, and 540 K (λ_*m*_ = *T*_*ref*_/*T*_*m*_). We used one temperature of 280 K and 9 discrete temperatures exponentially distributed from 300 to 540 K.

For comparison, ST simulations of CLN025 and Trp-Cage in unfolded and folded initial conformations (*U*_*ST*_ and *F*_*ST*_) were performed with 20 temperature rungs ranging from 280.0 to 500.0 K. As shown in ref 18, the solute–solute temperature in SST2 simulations is not the effective temperature of the solute, as the solvent is kept at a reference temperature and the solute–solvent interactions are scaled by $\sqrt{\lambda_{m}}$ . The effective solute temperature results from the balance between the two scaled energies, *E*_*pp*_ and *E*_*pw*_. The temperature ranges in ST and SST2 were chosen to approximate the two extreme temperatures in the stability curve for CLN025 and Trp-Cage (folded fractions of around 1.0 and 0.0, respectively), which explains the difference in the range of temperature between the ST and SST2 simulations.

The effect of changing the reference temperature to a higher value was investigated for CLN025 and Trp-Cage. SST2 simulations of CLN025 and Trp-Cage in the folded states were performed with 10 λ rungs, ranging from 280.0 to 540.0 K with *T*_*ref*_ = 350 K, resulting in λ values of 1.25, 1.16, 1.08, 1.0, 0.93, 0.87, 0.81, 0.75, 0.70, and 0.65, corresponding to solute–solute equivalent temperatures of 280.0, 301.6, 324.9, 350, 376.2, 404.4, 434.7, 467.3, 502.3, and 540 K. Note that if the solute–solute corresponding temperature distributions are similar at *T*_*ref*_ = 300 K and *T*_*ref*_ = 350 K, the temperature of the solvent is simulated at the reference temperature, resulting in a 50 K temperature difference.

Given the size of protein–peptide complexes, during a classical ST simulation, we would need to use an excessive number of ladders to ensure a good exchange rate. Consequently, as the temperature increases, the receptor is likely to unfold, and adding positional restraints to maintain the receptor structure could be required. For this reason, we chose to use only the SST2 algorithm to simulate the p97/PNGase complex. In these simulations, only the peptide ligand was considered as part of the solute subject to scaling, while the receptor remained unscaled. Importantly, no constraints were applied to either the receptor or ligand, allowing the system to evolve freely under the SST2 framework.

To assess SST2’s performance in simulating protein–peptide interactions, we performed eight molecular dynamics (MD) simulations of the p97/PNGase complex of 20 μs, four starting from the folded state and four starting from the unfolded state in an explicit solvent (Table 1). To increase the sampling efficiency, we used a reference temperature *T_ref_* = 320 K, resulting in 10 λ rungs of 1.14, 1.00, 0.91, 0.82, 0.75, 0.68, 0.07, 0.56, 0.50, and 0.46, corresponding to solute–solute temperatures of 280.0, 320.0, 352.9, 389.2, 429.2, 473.3, 521.9, 575.6, 634.8, and 700 K. Preliminary tests indicated that a maximum solute–solute temperature of 700 K was optimal for the system, allowing us to obtain a corrected maximum temperature of 500 K. At this temperature, the binding percentage was observed to be close to 0%, providing an ideal condition for evaluating the performance of the algorithm.

#### Exclusion of Proline ω Dihedral Angles

As will be shown later, CLN025 proline 4 in the *cis* conformation can trap the protein in an unfolded state. To avoid this issue, an additional option was added to the SST2 script to exclude proline ω dihedral angles from the solute-scaled intramolecular energy term *E*_*pp*_^(1)^ and keep them in the unscaled solute intramolecular energy term *E*_*pp*_^(2)^. To do this, all dihedral terms containing atoms *N*_(*i*)_ and *C*_(*i*–1)_ (where (*i*) indicates the proline residue number) at positions 2 and 3 of the dihedral term were excluded from *E*_*pp*_^(1)^ and kept in *E*_*pp*_^(2)^. This option was enabled on replicas 3 and 4 of Trp-Cage *F* and *U* simulations, and on all replicas of the Trp-Cage *F*_350 K_ simulations (Table 1).

### REST2 Simulations

To conduct REST2 simulations, we used the Psivant implementation of REST2 in OpenMM. The femto package was taken from github.com/Psivant/femto. The same protocol for system preparation was used as in SST2, and a 2 ps interval was used to attempt a lambda swap between neighboring lambdas. Ten lambdas were used with the same temperatures as those used in the SST2 simulations. For CLN025, 4 replicas of 1.0 μs were run starting from the folded and unfolded forms. For the TrpCage, 4 replicas of 2.0 μs were run starting from the folded and unfolded forms, as for p97/PNGase complex (only the peptide ligand was considered as solute), 4 replicas of 1.0 μs were ran starting from bounded and unbounded forms (Table 1). The first 10% of the simulations were removed for the analysis.

### Analysis

Structural alignment and Root Mean Square Deviation (RMSD) were computed on the backbone atoms by removing the first and last residues of chignolin (residues 2 to 9 were used) and the first two and last two residues of Trp-Cage (residues 3 to 18 were used). In the case of the p97/PNGase complex, trajectory alignment was performed on the PNGase backbone atoms, and the RMSD was computed using all the backbone atoms of p97.

In order to gain a detailed understanding of the p97 peptide conformation within the PNGase binding site, Principal Component Analysis (PCA) was conducted on simulation frames with a peptide RMSD of less than 1.0 nm. Subsequently, the resulting peptide conformations were clustered using the HDBSCAN algorithm^51^ on the first four components.

The MDAnalysis^52^ Python package was used together with some homemade Python scripts to analyze the MD simulations and perform statistical analysis and plotting.

The Python package scipy^53^ was used to compute four-parameter logistic (4PL) regression on the folding fraction as a function of temperature curves to extract the melting temperature. The equation model used is

19 $$y=d+\frac{a-d}{1+{\left(x/c\right)}^{b}}$$

where *a* is the folded fraction at low temperature, *d* is the folded fraction at high temperature, *c* is the inflection point, also called the melting temperature *T*_*m*_, and *b* is the Hill slope of the curve (or the steepness of the curve at *T*_*m*_).

## Supplementary Material



## Data Availability

The various Python scripts used in this work are available on GitHub at github.com/samuelmurail/SST2. Simulation trajectories have been deposited at zenodo.org/records/13772542 with the doi 10.5281/zenodo.13772541.

## Notes

Simulation details; discussion of temperature swap interval; analysis of SST2 computing performance; and convergence of ST, SST1, REST2, and SST2 simulations (PDF)

## References

[1] Jumper J., Evans R., Pritzel A. (2021). Highly accurate protein
structure prediction with AlphaFold. Nature.

[2] Mirdita M., Schütze K., Moriwaki Y., Heo L., Ovchinnikov S., Steinegger M. (2022). ColabFold: making protein folding accessible to all. Nat. Methods.

[3] Evans, R. Protein complex prediction with AlphaFold-Multimer, bioRxiv, 2022.

[4] Johansson-Åkhe I., Wallner B. (2022). Improving peptide-protein docking with AlphaFold-Multimer
using forced sampling. Front. Bioinformatics.

[5] Bret H., Gao J., Zea D. J., Andreani J., Guerois R. (2024). From interaction networks
to interfaces, scanning intrinsically disordered regions using AlphaFold2. Nat. Commun..

[6] Adcock S. A., McCammon J. A. (2006). Molecular Dynamics:
Survey of Methods for Simulating
the Activity of Proteins. Chem. Rev..

[7] Klepeis J. L., Lindorff-Larsen K., Dror R. O., Shaw D. E. (2009). Long-Timescale Molecular
Dynamics Simulations of Protein Structure and Function. Curr. Opin. Struct. Biol..

[8] Hansson T., Oostenbrink C., van Gunsteren W. (2002). Molecular Dynamics Simulations. Curr. Opin. Struct. Biol..

[9] Sugita Y., Okamoto Y. (1999). Replica-exchange molecular dynamics
method for protein
folding. Chem. Phys. Lett..

[10] Marinari E., Parisi G. (1992). Simulated Tempering:
A New Monte Carlo Scheme. Europhys. Lett..

[11] Lyubartsev A. P., Martsinovski A. A., Shevkunov S. V., Vorontsov-Velyaminov P. N. (1992). New approach
to Monte Carlo calculation of the free energy: Method of expanded
ensembles. J. Chem. Phys..

[12] Liu P., Kim B., Friesner R. A., Berne B. J. (2005). Replica Exchange with Solute Tempering:
A Method for Sampling Biological Systems in Explicit Water. Proc. Natl. Acad. Sci. U.S.A..

[13] Huang X., Hagen M., Kim B., Friesner R. A., Zhou R., Berne B. J. (2007). Replica exchange
with solute tempering: efficiency
in large scale systems. J. Phys. Chem. B.

[14] Wang L., Friesner R. A., Berne B. J. (2011). Replica
Exchange with Solute Scaling:
A More Efficient Version of Replica Exchange with Solute Tempering
(REST2). J. Phys. Chem. B.

[15] Mori T., Miyashita N., Im W., Feig M., Sugita Y. (2016). Molecular
Dynamics Simulations of Biological Membranes and Membrane Proteins
Using Enhanced Conformational Sampling Algorithms. Biochimica et Biophysica Acta (BBA) - Biomembranes.

[16] Harpole T. J., Delemotte L. (2018). Conformational
Landscapes of Membrane Proteins Delineated
by Enhanced Sampling Molecular Dynamics Simulations. Biochimica et Biophysica Acta (BBA) - Biomembranes.

[17] Heo L., Sugita Y., Feig M. (2022). Protein Assembly
and Crowding Simulations. Curr. Opin. Struct.
Biol..

[18] Stirnemann G., Sterpone F. (2015). Recovering Protein
Thermal Stability Using All-Atom
Hamiltonian Replica-Exchange Simulations in Explicit Solvent. J. Chem. Theory Comput..

[19] Jo S., Jiang W. (2015). A generic implementation of replica exchange with solute
tempering
(REST2) algorithm in NAMD for complex biophysical simulations. Comput. Phys. Commun..

[20] Bussi G. (2014). Hamiltonian
replica exchange in GROMACS: a flexible implementation. Mol. Phys..

[21] Zhang C., Ma J. (2008). Comparison of Sampling Efficiency
between Simulated Tempering and
Replica Exchange. J. Chem. Phys..

[22] Rosta E., Hummer G. (2010). Error and efficiency of simulated tempering simulations. J. Chem. Phys..

[23] Denschlag R., Lingenheil M., Tavan P., Mathias G. (2009). Simulated Solute Tempering. J. Chem. Theory Comput..

[24] McKiernan K. A., Husic B. E., Pande V. S. (2017). Modeling the mechanism of CLN025
beta-hairpin formation. J. Chem. Phys..

[25] Honda S., Akiba T., Kato Y. S., Sawada Y., Sekijima M., Ishimura M., Ooishi A., Watanabe H., Odahara T., Harata K. (2008). Crystal Structure of a Ten-Amino Acid Protein. J. Am. Chem. Soc..

[26] Hornak V., Abel R., Okur A., Strockbine B., Roitberg A., Simmerling C. (2006). Comparison of multiple Amber force
fields and development of improved protein backbone parameters. Proteins.

[27] Day R., Paschek D., Garcia A. E. (2010). Microsecond simulations of the folding/unfolding
thermodynamics of the Trp-cage miniprotein. Proteins.

[28] Pan A. C., Weinreich T. M., Piana S., Shaw D. E. (2016). Demonstrating an
Order-of-Magnitude Sampling Enhancement in Molecular Dynamics Simulations
of Complex Protein Systems. J. Chem. Theory
Comput..

[29] Neidigh J. W., Fesinmeyer R. M., Andersen N. H. (2002). Designing a 20-Residue Protein. Nat. Struct. Biol..

[30] Honda S., Akiba T., Kato Y. S., Sawada Y., Sekijima M., Ishimura M., Ooishi A., Watanabe H., Odahara T., Harata K. (2008). Crystal Structure of a Ten-Amino Acid Protein. J. Am. Chem. Soc..

[31] Zhao G., Zhou X., Wang L., Li G., Schindelin H., Lennarz W. J. (2007). Studies on peptide:N-glycanase-p97 interaction suggest
that p97 phosphorylation modulates endoplasmic reticulum-associated
degradation. Proc. Natl. Acad. Sci. U.S.A..

[32] Maier J. A., Martinez C., Kasavajhala K., Wickstrom L., Hauser K. E., Simmerling C. (2015). ff14SB: Improving
the Accuracy of
Protein Side Chain and Backbone Parameters from ff99SB. J. Chem. Theory Comput..

[33] Nguyen P. H., Okamoto Y., Derreumaux P. (2013). Communication:
Simulated Tempering
with Fast on-the-Fly Weight Determination. J.
Chem. Phys..

[34] Park S., Pande V. S. (2007). Choosing Weights
for Simulated Tempering. Phys. Rev. E.

[35] Timr S., Sterpone F. (2021). Stabilizing or Destabilizing:
Simulations of Chymotrypsin
Inhibitor 2 under Crowding Reveal Existence of a Crossover Temperature. J. Phys. Chem. Lett..

[36] Doshi U., Hamelberg D. (2009). Reoptimization of the AMBER Force
Field Parameters
for Peptide Bond (Omega) Torsions Using Accelerated Molecular Dynamics. J. Phys. Chem. B.

[37] Wakefield A. E., Wuest W. M., Voelz V. A. (2015). Molecular Simulation of Conformational
Pre-Organization in Cyclic RGD Peptides. J.
Chem. Inf. Model..

[38] Dai B., Chen J.-N., Zeng Q., Geng H., Wu Y.-D. (2024). Accurate
Structure Prediction for Cyclic Peptides Containing Proline Residues
with High-Temperature Molecular Dynamics. J.
Phys. Chem. B.

[39] Kamiya M., Sugita Y. (2018). Flexible Selection of the Solute
Region in Replica
Exchange with Solute Tempering: Application to Protein-Folding Simulations. J. Chem. Phys..

[40] Appadurai R., Nagesh J., Srivastava A. (2021). High resolution
ensemble description
of metamorphic and intrinsically disordered proteins using an efficient
hybrid parallel tempering scheme. Nat. Commun..

[41] Wang Y., Herron L., Tiwary P. (2022). From data
to noise to data for mixing
physics across temperatures with generative artificial intelligence. Proc. Natl. Acad. Sci. U.S.A..

[42] Benayad, Z. ; Stirnemann, G. Hamiltonian replica exchange augmented with diffusion-based generative models and importance sampling to assess biomolecular conformational basins and barriers. arXiv, 2025. 10.1021/acs.jctc.5c00738 41165096

[43] Moors S. L. C., Michielssens S., Ceulemans A. (2011). Improved Replica Exchange Method
for Native-State Protein Sampling. J. Chem.
Theory. Comput..

[44] Terakawa T., Kameda T., Takada S. (2011). On easy implementation of a variant
of the replica exchange with solute tempering in GROMACS. J. Comput. Chem..

[45] Eastman P., Swails J., Chodera J. D. (2017). OpenMM 7: Rapid Development
of High Performance Algorithms for Molecular Dynamics. PLoS Comput. Biol..

[46] Patriksson A., Spoel D. v. d. (2008). A temperature predictor for parallel
tempering simulations. Phys. Chem. Chem. Phys..

[47] Onufriev A., Bashford D., Case D. A. (2004). Exploring protein native states and
large-scale conformational changes with a modified generalized born
model. Proteins.

[48] Li D.-W., Brüschweiler R. (2010). NMR-Based
Protein Potentials. Angew. Chem., Int. Ed..

[49] Jorgensen W. L., Chandrasekhar J., Madura J. D., Impey R. W., Klein M. L. (1983). Comparison
of Simple Potential Functions for Simulating Liquid Water. J. Chem. Phys..

[50] Hopkins C. W., Le Grand S., Walker R. C., Roitberg A. E. (2015). Long-Time-Step Molecular
Dynamics through Hydrogen Mass Repartitioning. J. Chem. Theory Comput..

[51] McInnes, L. ; Healy, J. Accelerated Hierarchical Density Based Clustering. In IEEE International Conference on Data Mining Workshops (ICDMW), 2017; pp 33–42.

[52] Michaud-Agrawal N., Denning E. J., Woolf T. B., Beckstein O. (2011). MDAnalysis:
A toolkit for the analysis of molecular dynamics simulations. J. Comput. Chem..

[53] Virtanen P., Gommers R., Oliphant T. E. (2020). SciPy 1.0: fundamental
algorithms for scientific computing in Python. Nat. Methods.

